# A Compact Monopole Antenna for Underwater Acoustic Monitoring Beacons

**DOI:** 10.3390/s22218392

**Published:** 2022-11-01

**Authors:** Stefania Bucuci, Andreea Constantin, Mirel Paun, Marius N. Pastorcici, Razvan D. Tamas, Alin Danisor, Rodica Constantinescu

**Affiliations:** 1 Department of Electronics and Telecommunications, Constanta Maritime University, 900663 Constanta, Romania; 2 Department of Applied Electronics, University Politehnica of Bucharest, 061071 Bucharest, Romania

**Keywords:** monopole antenna, compact antenna, meander line antenna, genetic algorithm, environmental factors monitoring, underwater noise monitoring, internet of things

## Abstract

Protected wetlands such as deltas, lakes or rivers provide a sanctuary for many endangered species. In order to protect these areas from illegal human interventions, it is necessary to monitor the unauthorized entrance of motor boats. In order to mitigate such an impact, we have developed a network of floating beacons for underwater acoustic monitoring, using LoRa communication modules operating at 433 MHz. Such beacons should be equipped with compact antennas. In this paper, we use a genetic algorithm approach to design the compact, monopole antennas required for the beacons; size constraints would apply not only to the radiating element but also to the ground plane. Although the antenna input is unbalanced, such a small ground plane may yield common mode currents on the antenna feeder, which distort the radiation pattern of the antenna. In order to investigate the effect of the common mode currents, we developed a distance averaging method, while, for characterizing the antenna, we used a single-antenna method. For the experimental validation of the system in real conditions, a continuous monitoring of the lake was carried out. During the monitoring, multiple events generated by incursions of motor boats were successfully detected and recorded.

## 1. Introduction

Monitoring environmental factors is of growing importance for investigating and understanding the impact of anthropic activities on the flora and fauna. More than 70% of the Earth’s surface is covered by water; the underwater world hosts rich and unique ecosystems of great importance for humankind. The underwater world is not deaf, even if it would appear so. Water transport activities, or the exploitation of underwater resources, may aggress the fauna and induce irreversible losses [1,2]. 

The monitoring of underwater noise in research applications for the protection of the marine environment, with an emphasis on the state of the aquatic life, using beacons equipped with hydrophones has been successfully implemented in the project “Smart digital hydrophone system” in the region of Murcia, Spain. The hydrophone beacons deployed in the Mediterranean Sea near the Port of Cartagena are powered by solar cells and transmit data wirelessly to the ground station using a satellite connection [3].

A similar underwater monitoring system mounted on a buoy was deployed in the Miramare Marine Protected Area. The system contains several sensors and instruments for monitoring multiple water quality parameters, including a hydrophone deployed at a depth of 14 m. The beacon uses six solar panels for harvesting energy and two rechargeable lead batteries for storing it [4].

A water quality monitoring system in the form of an autonomous navigating floating platform was successfully implemented in the project “Drone on the Volga”. The system monitors water pollution in the Kuybyshev reservoir, the main drinking water source in the region. The system uses floating platforms, referred to as drones, powered by solar panels and batteries, fitted with sensors for temperature, pH, dissolved oxygen, water conductivity and NH4+ and NO3− ions. The transmission of collected data to the ground station is carried out through a 4G radio connection [5]. Similar platforms mounted on fixed buoys are deployed in the open waters of Alaska in an attempt to protect and preserve the habitat of the beluga [6], as well as in smart fisheries [7].

Following the analysis of the state-of-the-art solutions, it is observed that the most common source of energy for distributed sensor platforms is a combination of photovoltaic panels and batteries, as it is based on a mature technology that has been tested for a long time and has demonstrated its efficiency and durability. Other power systems such as micro wind turbines or systems that harness the energy of water currents are not used in sensor applications. For the radio link between the beacon and the ground station, satellite, 4G and LoRa are the preferred technologies.

We have developed a network of floating beacons as part of an Internet of Things (IoT) system for underwater acoustic monitoring in the Danube Delta area, a unique ecosystem in South-Eastern Europe. The beacons transmit underwater sound records to a remote data center by using LoRa communication modules. Data transmission is triggered whenever an underwater noise threshold is exceeded. Since the operating environment is rich in vegetation, and the transmission antennas on the beacons are close to the water surface, we have chosen the frequency range around 433 MHz in order to provide a good radio wave penetration. 

Such beacons should be equipped with compact antennas providing a quasi-omnidirectional coverage in a horizontal plane. The beacons are designed to be deployed close to navigation and fishing areas; they are unattended and should be observable as little as possible. Despite a quite long wavelength, the antennas should be compact enough so as to not be damaged, e.g., hung up by fishing ropes. It has been shown [8] that antenna design with genetic algorithms (GA) provides a good tradeoff between gain and size. 

Compact antennas are a suitable choice for applications requiring small-sized antennas such as IoT applications. Different types of antennas are used in order to achieve miniaturization. For low frequencies such as the LoRa communication frequencies of 433 MHz or 868 MHz, size reduction can be acquired using the variation of an inverted -F antenna (IFA) configuration with a square-spiral section and a rectangular patch element [9] or microstrip antennas with changeable geometry based on IFA, which are created to be more appealing as logos or figures [10]. Another novel shape of compact printed monopole UWB antennas presented in [11] is a square tilted frame monopole. For dual band applications, a compact patch antenna with paired L-shape slots [12] is proposed for on- and off-body communications in a wireless body area network or antennas consisting of radiating strips [13] that are suitable for wireless power transfer systems.

Genetic algorithms are an important tool in the design and optimization process of compact antennas. From the innovative design of a miniaturized evolved patch antenna whose radiation properties have been enhanced with a Split Ring Resonator (SRR) placed between the top and the ground plane [14] to antenna structures consisting of a tapered radiating element fed by a microstrip line [15], or microstrip patch antennas [16,17,18] with various shapes and configurations, the evolutionary computing algorithms based on the principles of genetics and natural selection are suitable for any problem. These are very general algorithms, so they work in any environment. Genetic algorithms are one of the best ways to solve a problem for which little information is known, representing a clever exploitation of a random search to find the optimal solution. As long as the requirements are well defined and the goal of the problem is clear, the genetic algorithm will generate a quality solution.

In this paper we used a GA approach to design a compact, monopole antenna for underwater acoustic monitoring beacons, using LoRa communication modules at 433 MHz. As opposed to our previous work [19,20,21,22], we adapted the GA approach for monopole antennas. That is, the finite-sized ground plane is taken into account in the simulations performed between two iterations. 

Moreover, here, we let the objective function essentially deal with only two characteristics, i.e., size and gain. Impedance matching is somewhat less important and would only slow down the convergence rate of the algorithm. A transmission line impedance matching circuit can be easily integrated post-optimization in a narrow-band, monopole antenna, as we further present in our paper. 

Standard gain probe antennas for low-frequency gain measurement often provide a low gain and a poor impedance matching. As a result, the traditional, two-antenna method might provide inaccurate results [23,24,25]. We therefore propose the use of a simple, single-antenna method [26,27,28] in order to characterize the compact monopole antenna. 

When using a monopole antenna for a small-sized beacon, size constraints would apply not only to the radiating element but also to the ground plane. As a result, the ground plane is electrically small at 433 MHz. Although the antenna input is unbalanced, such a small ground plane may yield common mode currents on the antenna feeder, which distort the radiation pattern of the antenna under test (AUT) [29,30,31].

Common mode currents usually appear on the outside conductor of the antenna feed line when a symmetrical antenna is fed through an asymmetrical line or when the ground plane is electrically small. The magnitude of these currents can even be close to the magnitude of the feed currents, and for this reason, the common mode currents can have a strong influence on the radiated field generated by the antenna–feed line set. Thus, when measuring the radiation of an antenna, it is very important to find solutions to reduce or even to cancel the effect of radiation from common mode currents. Among the most frequently used solutions to solve this inconvenience are: the use of baluns or a coil-shaped feed line [32,33] and the replacement of the coaxial cable with an optical fiber [34].

In order to investigate the effect of the common mode currents, we used a distance averaging approach [35]. 

The paper is organized as follows: the second section presents the beacon for underwater noise monitoring and the communication requirements; the third section focuses on the antenna design; the fourth section deals with the antenna characterization method; the fifth section presents the results of the experimental validation.

## 2. Communication Requirements for the Underwater Noise Monitoring Beacons

**Figure 1:**
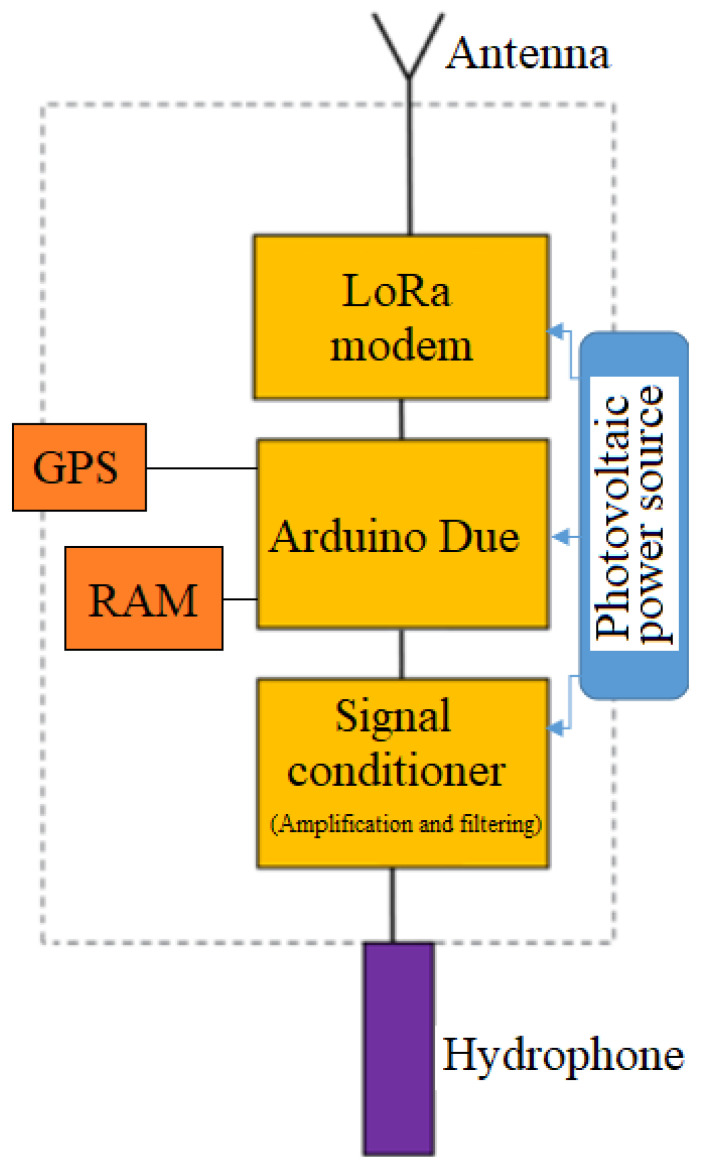
Block diagram of an underwater noise monitoring beacon.

The block diagram of the noise monitoring beacon is presented in Figure 1.

The sound transducer is a DolphinEar PRO-10 wideband (1–24 kHz) omnidirectional hydrophone [36]. The conditioning of the signal captured by the hydrophone is performed by means of a precision differential amplifier based on the INA333 op-amp. The local digital signal processing is implemented on an Arduino Due board. The radio link for communicating with the ground station is implemented using a LoRa RFM 98 modem operating at 433 MHz [37].

Since the internal RAM bank of the microcontroller on the Arduino Due board is insufficient to temporarily store enough acoustic signal samples until it is transmitted through the radio interface, an external 128 kB RAM bank was added.

In order to accurately determine the time of occurrence of an acoustic signal of interest, as well as to monitor the position of the beacon, an NEO-6M GPS module was employed [38].

The power source of the beacon is a photovoltaic system comprising a 10 W ETFE encapsulated solar panel, a buck-boost converter and a lead rechargeable battery. The measured current consumption of a beacon was 160 mA in stand-by and 180 mA when transmitting through LoRa. Presuming that the system is transmitting 25% of the total operating time, the estimated average consumption is 165 mA. Thus, a fully charged battery with a capacity of 5000 mAh would last 1.26 days. As a result, the daily consumption is 3960 mAh. The solar panel provides a nominal current of 2 A at 5 V. It is assumed that the system operating at 35% of its nominal capacity for an average of 6 h per day would result in a 4200 mAh charge per day, which would compensate for the diurnal discharge of the battery and thus allow for the uninterrupted operation of the beacon.

The program implemented on the beacon contains a trigger routine that continuously monitors the amplitude of the acoustic signal captured by the transducer and compares it with a threshold value. When this threshold is exceeded, the sound recording is initiated for a period of 5 s at a sampling rate of 12 kHz and 12 bits of resolution. The acquired acoustic signal is stored in the external RAM, the time and position information taken from the GPS is also included and then the information is sent through radio to the central node of the sensor network when the radio channel is available. Data transmission using the LoRa protocol is very slow; as a result, sending a 5 s record of an acoustic signal takes as long as 5 min. The low data rate does not impinge on the system performance since the monitored acoustic events are expected to have a low incidence rate (a couple of occurrences per day). Data transmission is fully encrypted using a Speck cipher with a 128-bit key. The beacon also has the ability to be triggered on demand by the user, if the environmental sound recording is desired at a certain moment, or to locate a lost beacon. The trigger threshold can also be changed from the central node by the user for each beacon individually.

**Figure 2:**
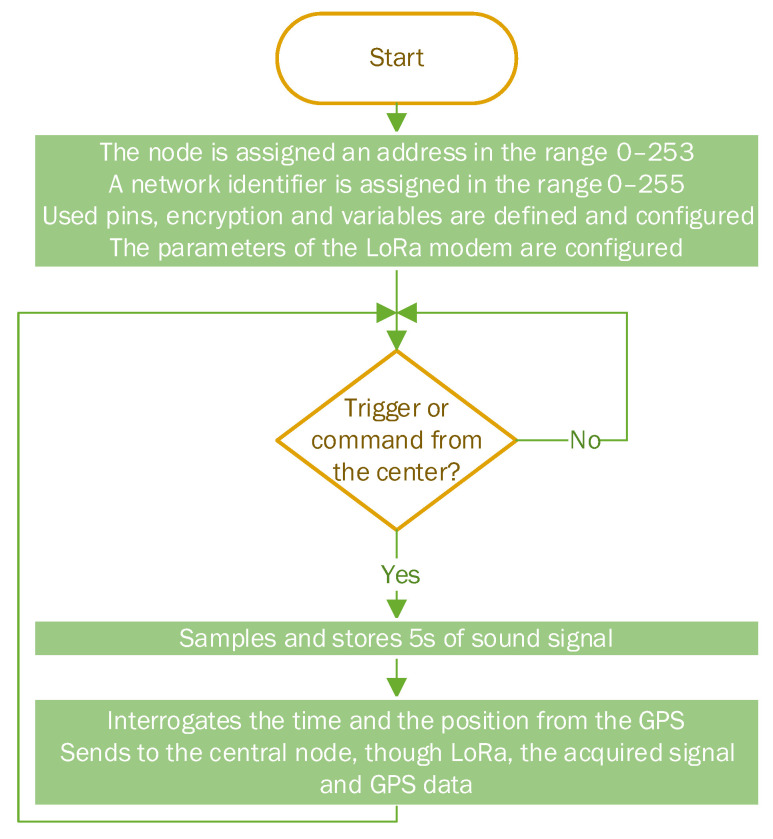
Beacon program flowchart.

**Figure 3:**
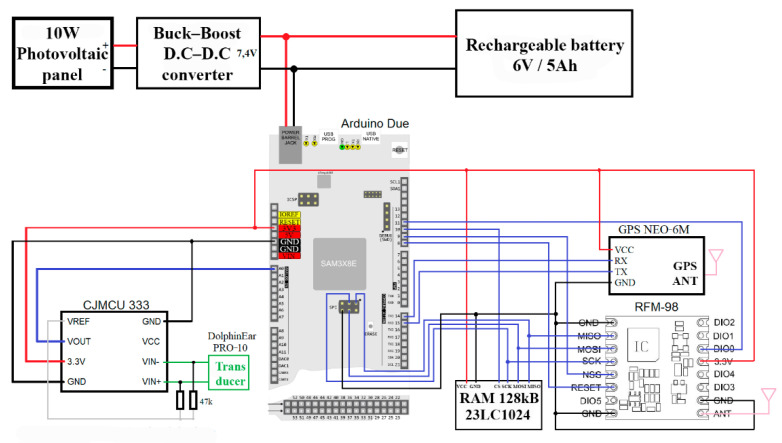
Electrical diagram of a beacon.

The program flowchart for the beacon is presented in Figure 2, and the electrical diagram is detailed in Figure 3. 

**Figure 4:**
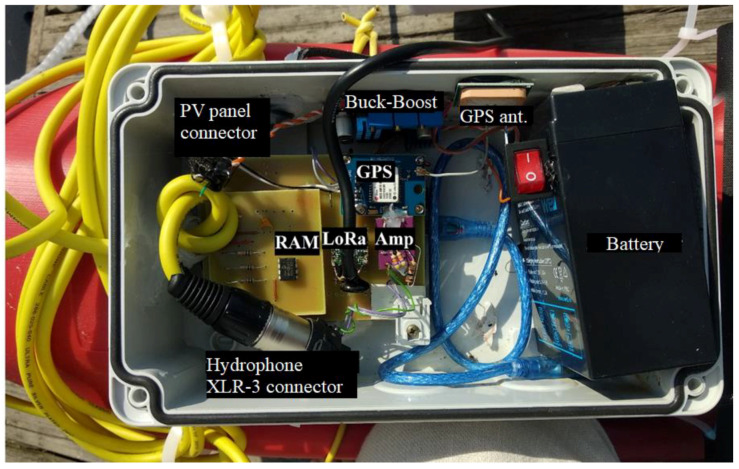
Electronic blocks on board a beacon.

The appearance of the physical implementation including all the electronic blocks is presented in Figure 4.

All connectors were sealed with silicone gaskets to prevent accidental water ingress. A ballast was attached to the hydrophone to keep it permanently submerged.

**Figure 5:**
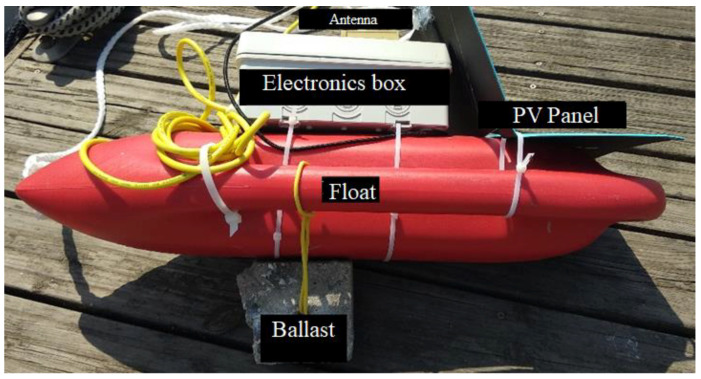
Complete and operational beacon.

In order to stabilize the beacon so as to keep the box containing the electronic blocks and the antenna upright, a ballast was mounted under the buoy, as shown in Figure 5.

**Figure 6:**
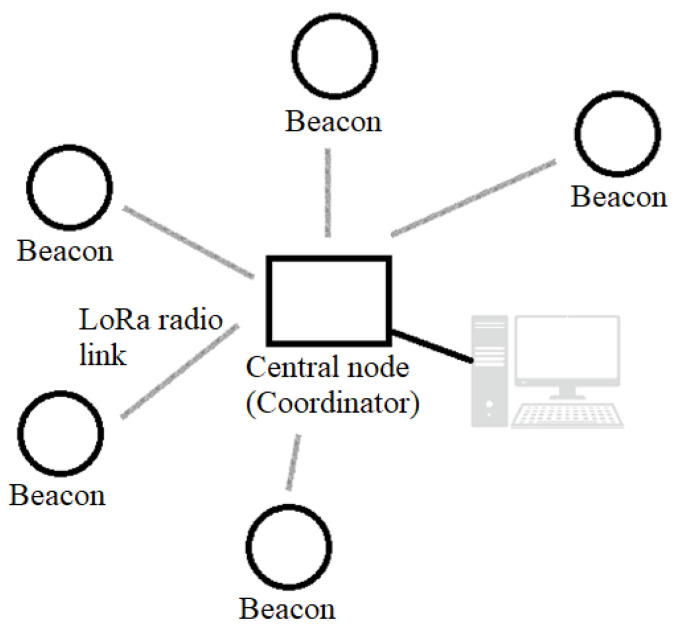
Beacon network configuration.

The beacon network was implemented in a star configuration. The central node or coordinator of the network is connected via a USB-emulated serial interface to a computer that stores the received data locally, as presented in Figure 6.

**Figure 7:**
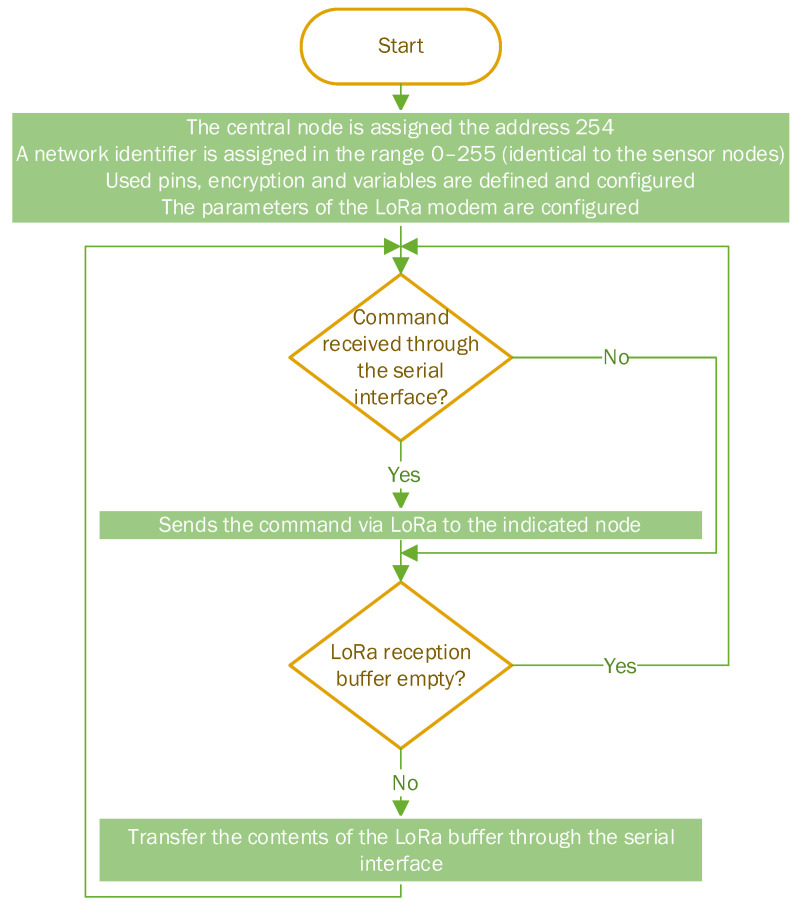
Central node program flowchart.

**Figure 8:**
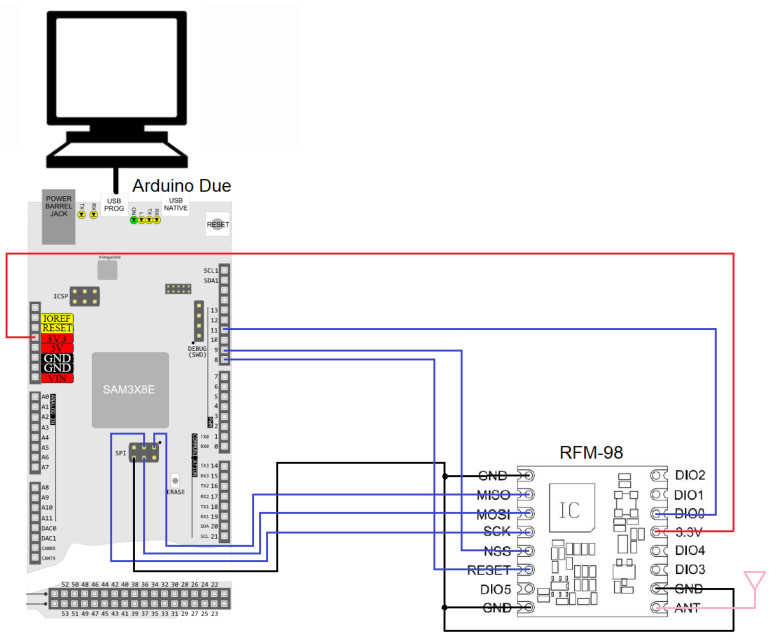
Electrical diagram of the central node.

**Figure 9:**
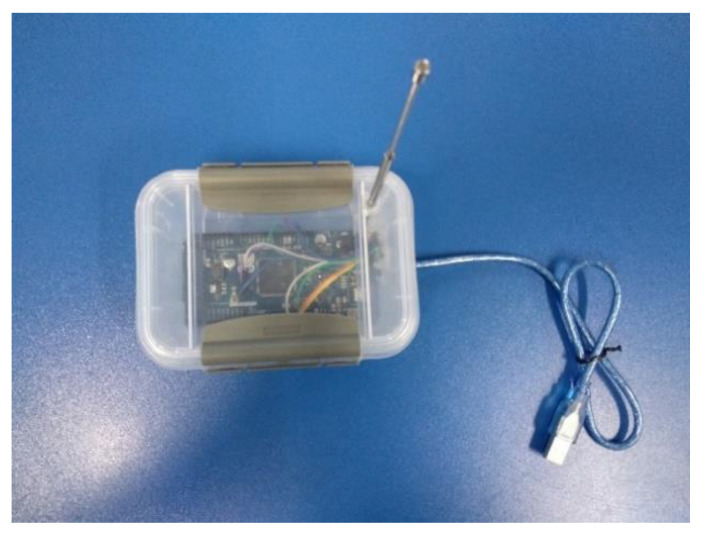
Central node.

The program flowchart for the central node is presented in Figure 7, and the electrical diagram and practical implementation are detailed in Figure 8 and Figure 9. The central node uses a simple omnidirectional monopole antenna, as there are no particular constrains.

The program for monitoring and storing the data received from the beacons was developed in the LabVIEW graphical programming environment and runs on the computer connected to the central node of the sensor network. The program allows for real-time monitoring of the beacons, automatic data storage and the modification and query of the trigger threshold for each beacon in the network individually.

**Figure 10:**
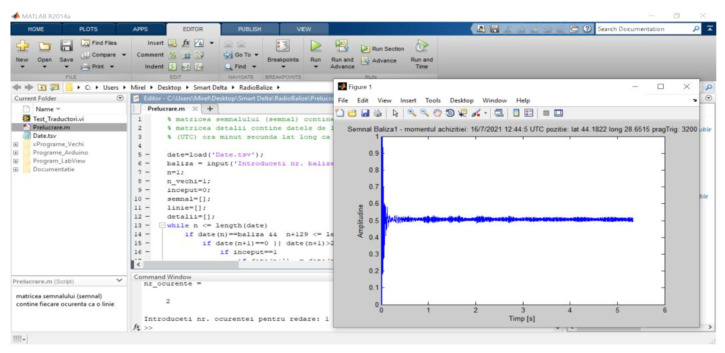
Selecting a signal from the stored database.

The data received from the beacons are stored on the computer hard disk as a tab-separated values (TSV) text file, referred to as the database, which contains the identification data of the beacon that provided the signal, the timestamp of the acquisition and the position of the beacon at the moment the signal was acquired. A MATLAB script was created for searching and processing the stored acoustic signal sequences. Figure 10 shows the effect of selecting the Beacon 1 first occurrence signal.

## 3. Antenna Design

In order to design a compact monopole antenna resonating at 433 MHz for the LoRa standard communication between the beacon and the remote data center, a genetic algorithm is used for miniaturization. A monopole antenna is resonating at the desired frequency when its length is one-quarter of the wavelength. Thus, the monopole antenna with a linear length of approximately 172.5 mm should be reduced to a miniaturized planar structure by folding the monopole into a meander line antenna (MLA). 

There are two types of MLAs—the uniform MLA, which presents equal-sized meanders, and the non-uniform MLA, where the length and width of the meanders vary throughout the structure. As for the complexity of the structure, the elemental antenna has only one turn, comprising six segments, as shown in [19].

We propose the use of a genetic algorithm in order to generate the structure of a meandered monopole antenna by following a tradeoff between size reduction and gain maximization. The height of a monopole antenna is related to the wavelength (a quarter wavelength in most cases). However, the gain highly depends on the ground plane size when both the ground length and width are less than 1.5λ. The goal of our GA design process is actually to lead to a radiating structure shorter than λ/4 by keeping the gain as close as possible to that provided by a full-size quarter wave monopole. 

**Figure 11:**
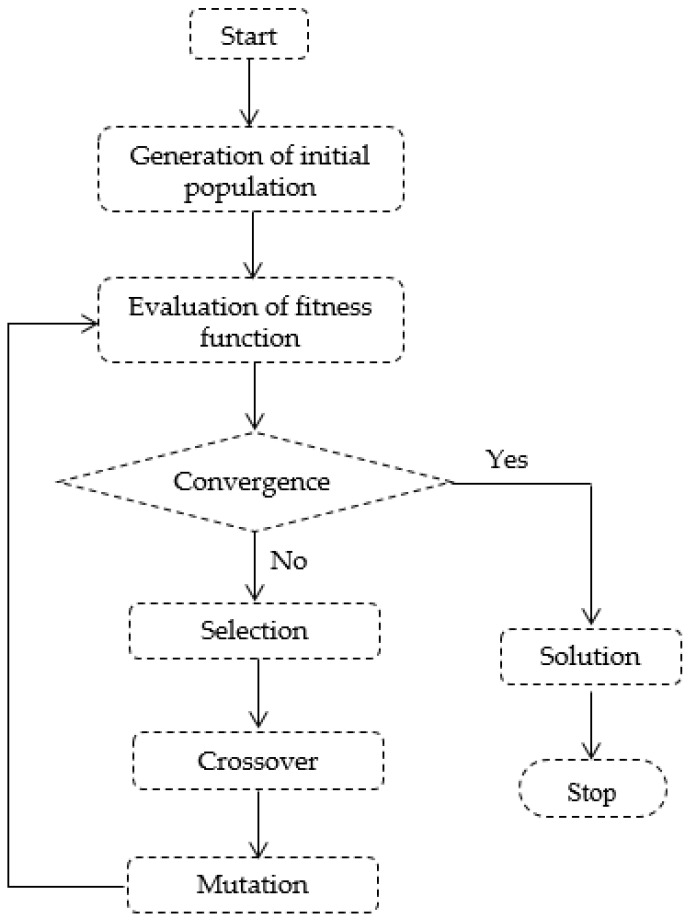
Flowchart of genetic algorithm.

Figure 11 presents the flowchart of the genetic algorithm. The initial population, i.e., generation zero, includes four antennas considered to be meander line antennas with a simple structure consisting of only one turn. As opposed to a random generation, we set the initial population to have four specific chromosomes with six genes each, in the interest of inheriting valuable characteristics. The corresponding length (in millimeters) of each segment that constitutes one turn of the meander line antenna is coded on six bits for each gene of the chromosomes.

The first chromosome is a quarter-wavelength monopole antenna; although it is the longest one, it yields the highest gain. This particular chromosome is coded as an MLA, with the horizontal segments equal to zero. The rest of the antennas from the initial population are meander monopole antennas with different length-to-width ratios for their segments, such as 1/1, 1/2 and 2/1, in order to give diversity to the population. All antennas from the initial population are designed to resonate at 433 MHz. 

After the generation of the initial population, the next step is to evaluate the fitness function for each *i*-th antenna at every *p*-th iteration of the algorithm:

(1) $$f^{\left(p\right)}\left(G_{i},H_{i}\right)=r_{1}\cdot \frac{G_{i}}{G_{0}}+r_{2}\cdot \frac{H_{max}}{H_{i}},$$

where $G_{i}$ is the gain of the *i*-th antenna of the population, $G_{0}$ is the maximum gain figure corresponding to a straight monopole antenna, $H_{max}$ is the maximum height of the antenna set to 65 mm, $H_{i}$ is the height of the *i*-th antenna of the population and $r_{1}$ and $r_{2}$ are the weights depending on the importance of the optimization objective, set to 0.25 and 0.75, respectively. 

The convergence criterion of the algorithm is the fitness function value which must be close to 1 in order to obtain an MLA with a size of 65 mm by 65 mm and a maximum possible gain figure. As the algorithm converges, the *i*-th population becomes the optimal solution. Otherwise, the next step of the genetic algorithm is to implement the genetic operators, i.e., selection, crossover and mutation.

The selection operator sorts the chromosomes by the value of the fitness function in order to replace the least fit chromosome with the fittest, creating a pair of chromosomes with good genes to be inherited. Afterwards, the crossover operator replaces one-half of the remaining least fit chromosome with one-half of the fittest one to create new offspring. Lastly, the mutation randomly changes the value of one bit in the least fit three chromosomes to maintain diversity and to prevent premature convergence. Therefore, a new population of chromosomes is formed and has to be evaluated. 

An important stage of the design process using a genetic algorithm towards creating a new structure of a meander line monopole antenna is the electromagnetic simulation of each antenna for every new generation in order to obtain the $G_{i}$ and $H_{i}$ values for the fitness function of each chromosome before continuing with the next iteration. The simulation takes into account the small electric size of the ground plane, i.e., 0.17λ by 0.26λ, resulting from mounting constraints (the ground plane should fit into the lid of the box containing the electronic blocks). Another important optimization objective focuses on the input reactance of the antenna. However, this criterion was not included in the fitness function, as each antenna was scaled in the simulation in order to maintain the resonance at the frequency of interest.

**Figure 12:**
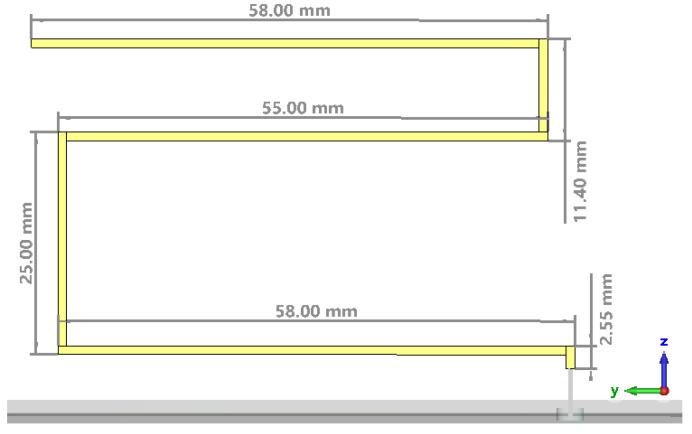
Details of the radiating element for the optimal structure.

As each new population is characterized regarding the antenna gain and height, the fitness function is evaluated, and after enough iterations, the convergence criterion would be met in order to obtain the final population of antennas. The details of the radiating element for the optimal structure are presented in Figure 12. 

**Figure 13:**
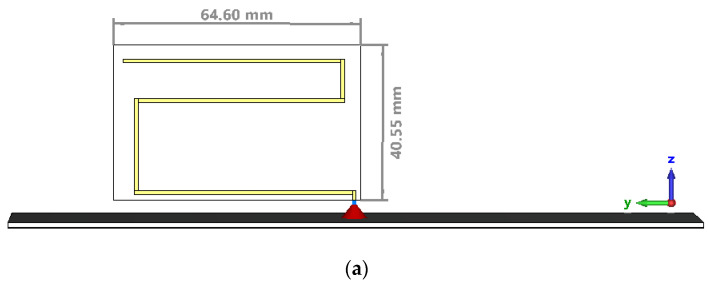

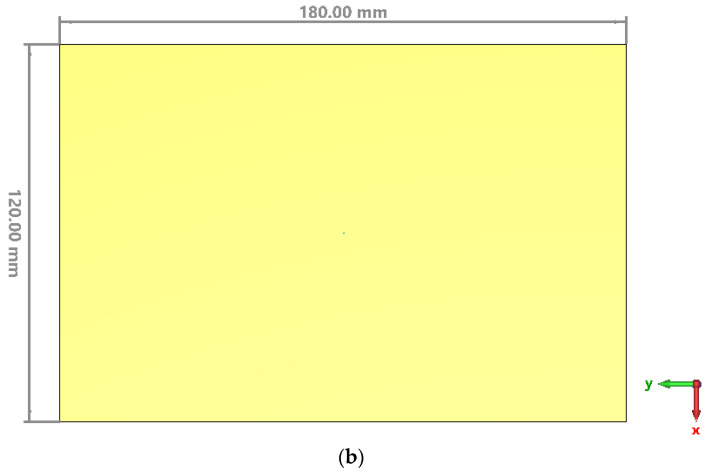
Full simulation model of the meander line monopole antenna: (**a**) Side view; (**b**) Top view—ground plane.

The optimal antenna was printed on an FR4 substrate that was 40.55 mm by 64.6 mm in size and 0.5 mm in width; the ground plane was printed on an FR4 substrate that was 120 mm by 180 mm in size and 1.5 mm in width. The conductor has a width of 1 mm, while the thickness is 35 µm. Figure 13 presents the full simulation model for the CST Microwave Studio of the meander line monopole antenna. A discrete port was used in the simulation as a feeding structure. 

**Figure 14:**
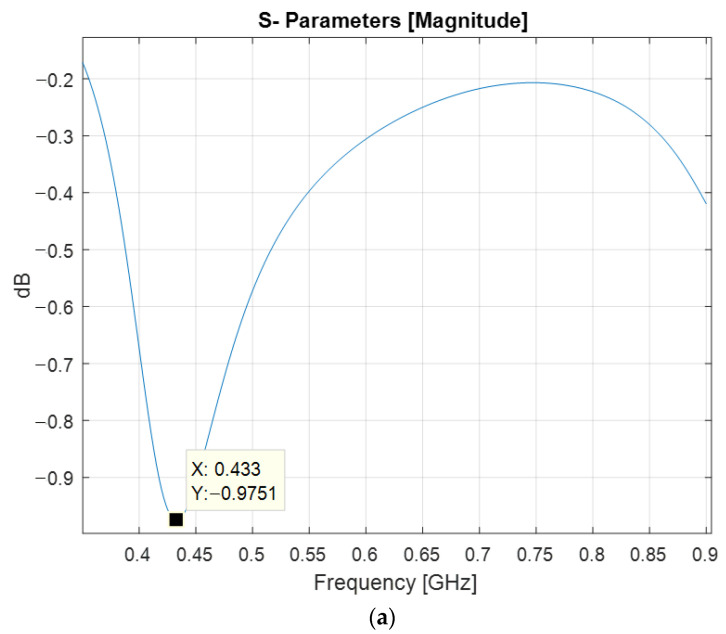

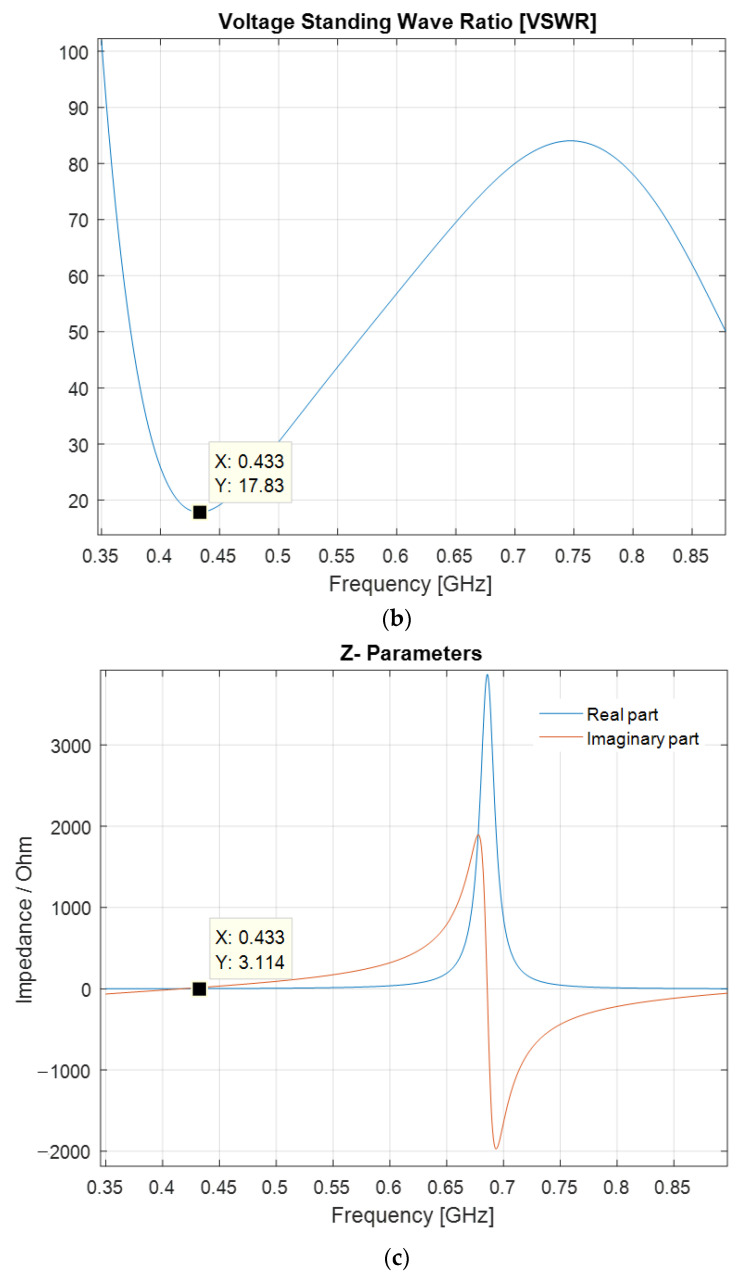
Impedance matching figures at the antenna input: (**a**) Reflection coefficient; (**b**) Voltage standing wave ratio; (**c**) Input impedance.

The magnitude of the input reflection coefficient, shown in Figure 14, has a minimum of −0.97 dB at 433 MHz, and the voltage standing wave ratio has a dip of 17.83 at the same frequency. The real part of the input impedance of the printed meander line monopole antenna presents a radiation resistance of 3.11 Ω. Since the normalizing impedance at the excitation port is 50 Ω, the impedance matching can be obtained by using a transmission line impedance matching circuit.

The input impedance of the antenna at the resonant frequency is purely resistive. The matching of a purely resistive load to an input port with the normalizing impedance of 50 Ω can be achieved by using an impedance inverter, i.e., a quarter-wavelength transmission line stub. The input impedance $Z_{in}$ is inversely proportional to the load resistance, $R_{a}$. This property allows for the transformation of a real load impedance as follows:

(2) $$Z_{in}=\frac{Z_{c}^{2}}{R_{a}}.$$

**Figure 15:**
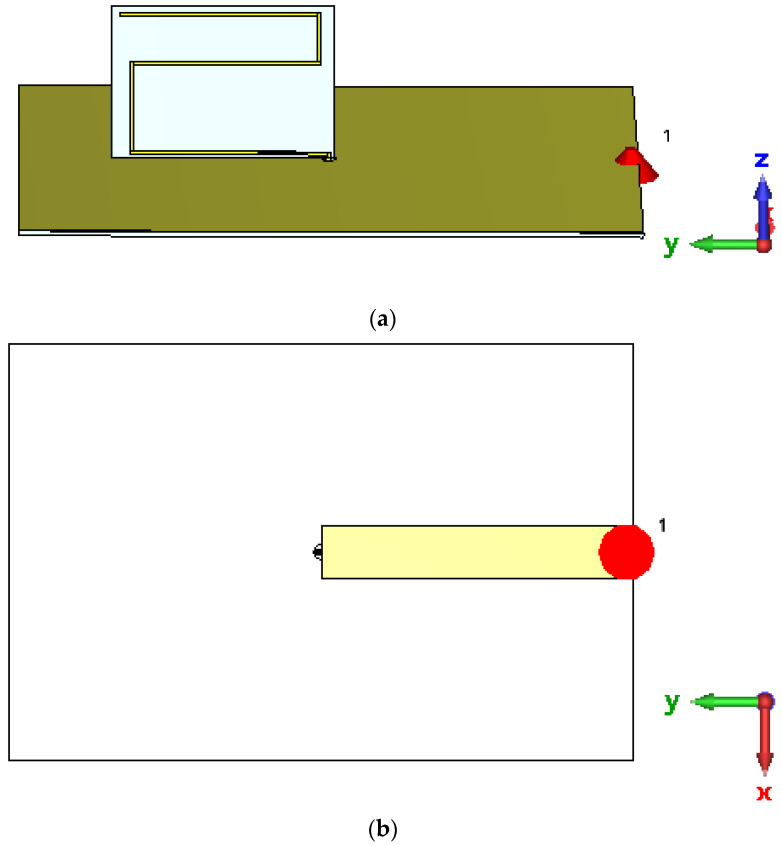
Simulation model of the meander line monopole antenna with an impedance matching circuit: (**a**) Overview; (**b**) Bottom view.

As a result, a transmission line with a characteristic impedance of 13.96 Ω is needed. Such a transmission line can be implemented in microstrip technology; the antenna and the microstrip line share the same ground plane, that is, the line conductor is printed on the lower side of the PCB (Figure 15). The above characteristic impedance requires a line conductor that is 16.08 mm by 88.98 mm in size on a FR4 substrate.

**Figure 16:**
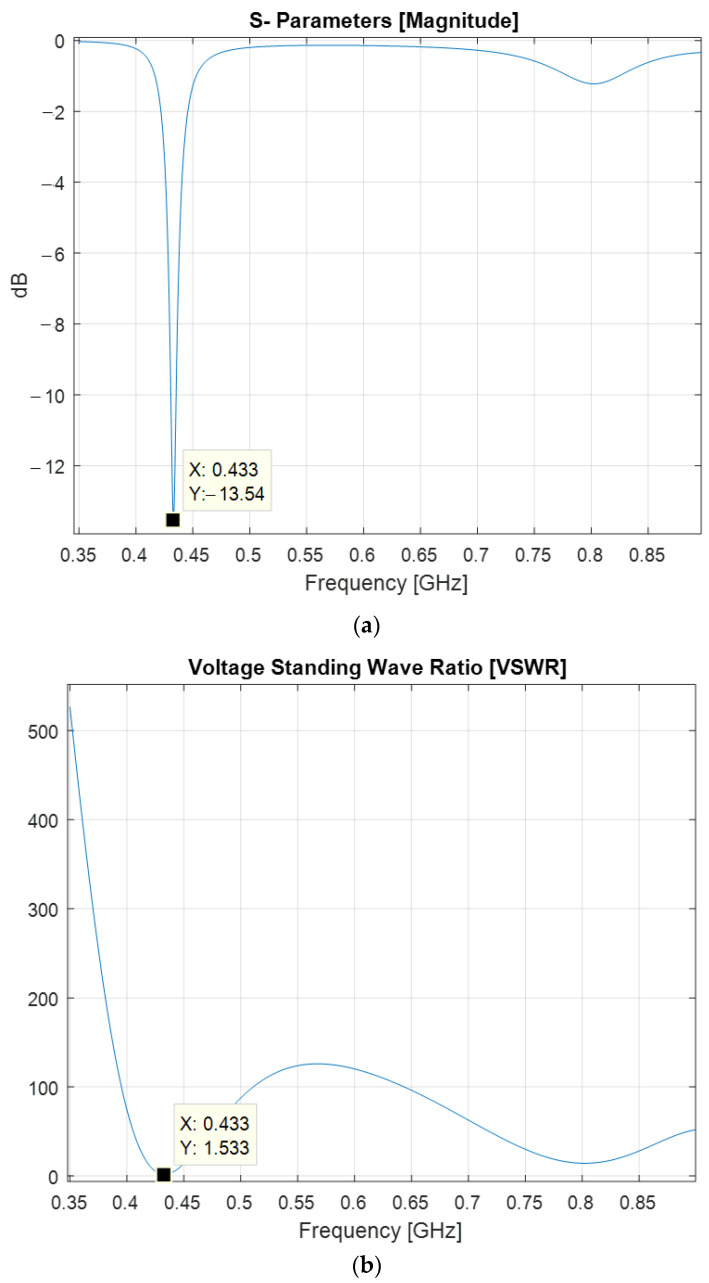
Impedance matching figures at the antenna input with an impedance matching circuit: (**a**) Reflection coefficient; (**b**) Voltage standing wave ratio.

With the input impedance matching circuit, the input reflection coefficient, shown in Figure 16, exhibits a lower magnitude of −13.54 dB at the frequency of interest, and the VSWR decreased down to 1.53 at the same frequency.

**Figure 17:**
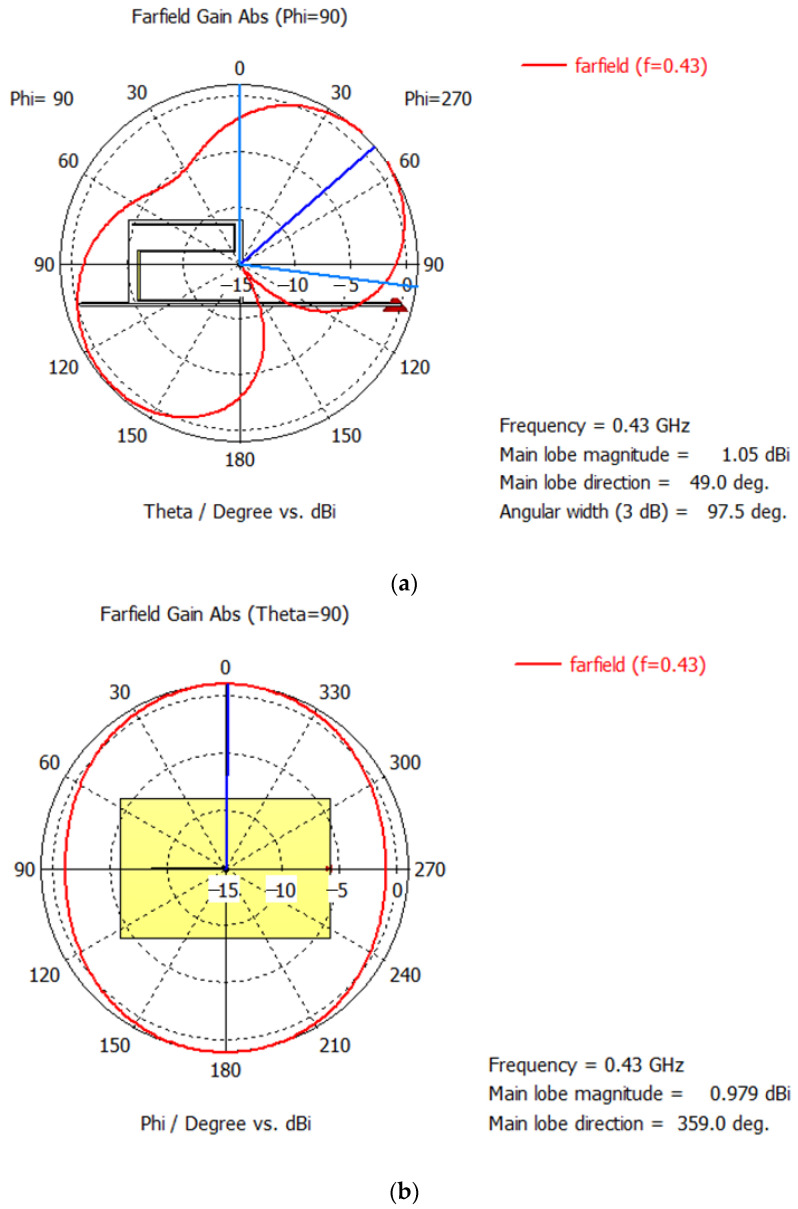
Radiation pattern of the printed meander line monopole antenna with an input impedance matching circuit: (**a**) E plane; (**b**) H plane.

Figure 17 presents the 2D radiation pattern of the printed meander line monopole antenna with an input impedance matching circuit. In the E plane, the main lobe direction is 49 deg., the main lobe magnitude is 1.05 dBi and the half-power beamwidth is 97.5 deg. The E-plane radiation pattern in Figure 17 was simulated under a free space assumption. Nevertheless, in a real environment, the antenna is placed at around 15 cm above the water surface. In that case, the radiation from the lobe pointing at 131 deg. to the water surface will be reflected at approximately −49 deg., therefore providing efficient communication in that direction as well. The H plane radiation pattern is omnidirectional. 

**Figure 18:**
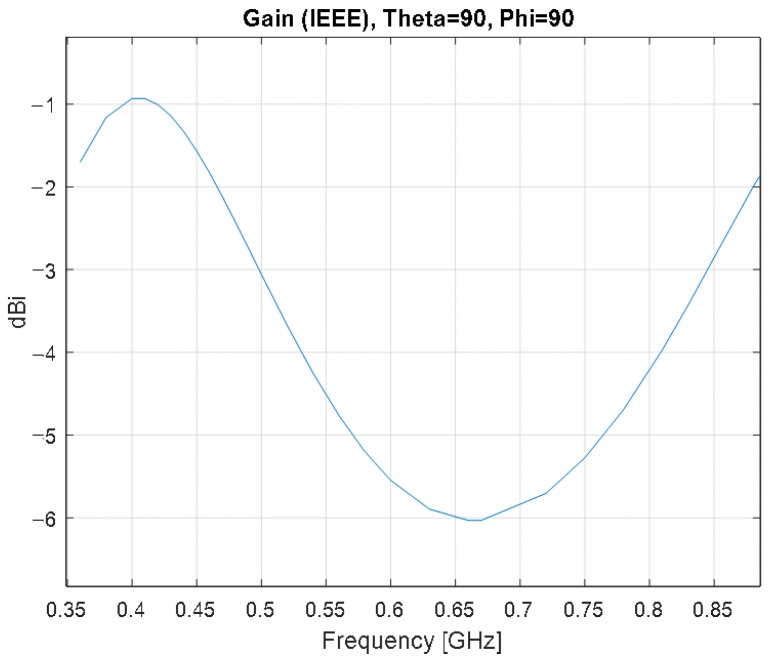
Gain variation over frequency for the simulation model of the meander line monopole antenna with impedance matching at θ=90 deg. and φ=90 deg.

Figure 18 presents the simulated gain variation over frequency for the simulation model of the meander line monopole antenna with impedance matching at θ=90 deg. and φ=90 deg.

As the frequency increases, the gain has an increasing trend as well. Around the frequency of interest, the gain is −1.2 dBi.

## 4. Antenna Characterization

Antenna gain measurements at low frequencies (typically in the VHF and UHF ranges) require large probe antennas in order to provide good radiation efficiency and input matching. As a result, the anechoic chamber should be physically large as well to comply with the far field requirements. Smaller probe antennas for V/UHF can be used only for measuring directive antennas; otherwise, the signal-to-noise ratio would lead to inaccurate results. 

In order to determine the gain of the meander line monopole antenna, we therefore applied a single-antenna method, as it does not require a probe antenna. The method consists of placing the antenna under test in front of a planar reflector, and it is based on a virtual transfer between the antenna and its image. Such a technique usually provides a good accuracy around the resonant frequency where the antenna provides a low input reflection coefficient. 

**Figure 19:**
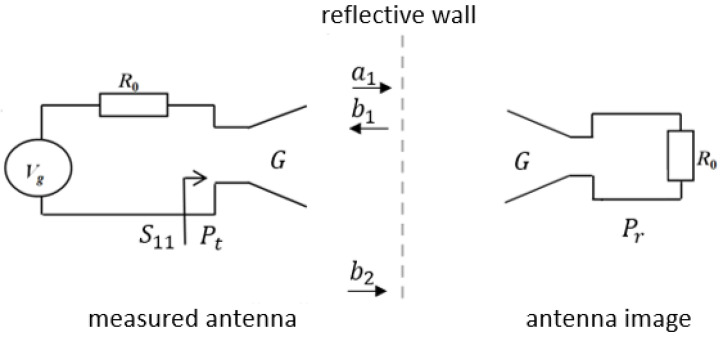
Measurement configuration for the single-antenna method.

The measurement configuration is shown in Figure 19. 

The Friis transmission formula for free-space can be adapted for the single-antenna method, as follows:

(3) $$\frac{P_{r}}{P_{t}}=G^{2}{\left(\frac{\lambda }{4\pi r}\right)}^{2},$$

where $P_{r}$ is the received power, $P_{t}$ is the transmitted power, G is the gain of the antenna, λ is the wavelength and r is two times the distance between the antenna and the reflector.

The power ratio can be written in terms of *S* parameters as

(4) $$\frac{P_{r}}{P_{t}}=\frac{R_{0}}{R_{a}\left(f\right)}\frac{{\left|S_{21}\right|}^{2}}{{\left|1-S_{11}\right|}^{2}\left(1-{\left|S_{11}\right|}^{2}\right)},$$

where $R_{0}$ is the normalizing impedance, $R_{a}\left(f\right)$ is the radiation resistance and $S_{11}$ is the reflection coefficient of the measured antenna.

The transfer function, $S_{21}$, is determined as the difference between the reflection coefficients of the antenna measured with and without the reflector:

(5) $$S_{21}=S_{11}^{refl.wall}-S_{11}^{anechoic}.$$

The gain of the measured meander line monopole antenna can then be expressed as:

(6) $$G=\sqrt{{\left(\frac{4\pi r}{\lambda }\right)}^{2}\frac{R_{0}}{R_{a}\left(f\right)}\frac{{\left|S_{21}\right|}^{2}}{{\left|1-S_{11}\right|}^{2}\left(1-{\left|S_{11}\right|}^{2}\right)}}.$$

Gain measurements on monopole antennas with an electrically small ground plane should also address the effect of the common mode currents that normally occur on the outer conductor of the feed line. 

In a previous paper [35], we focused on developing a method to reduce the effect of common currents but also on proposing a differential approach to extract the radiation generated by these currents from the total radiated field. The method that allows for a reduction of the effect of common mode currents, i.e., the distance averaging method, provides very good results; this is due to the fact that these currents have a distance variant distribution. The differential approach consists of proposing two measuring setups that are established according to the position of the probe antenna (PA) with respect to the feed line. 

**Figure 20:**
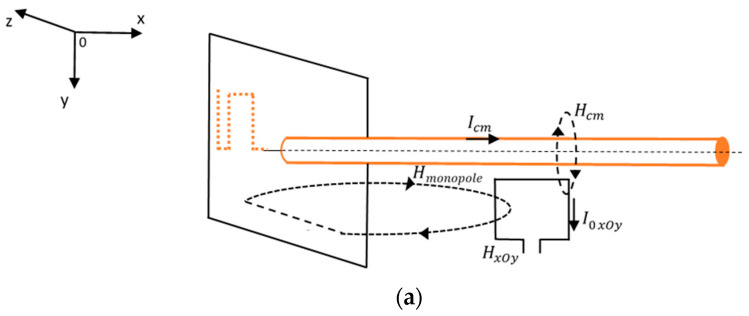

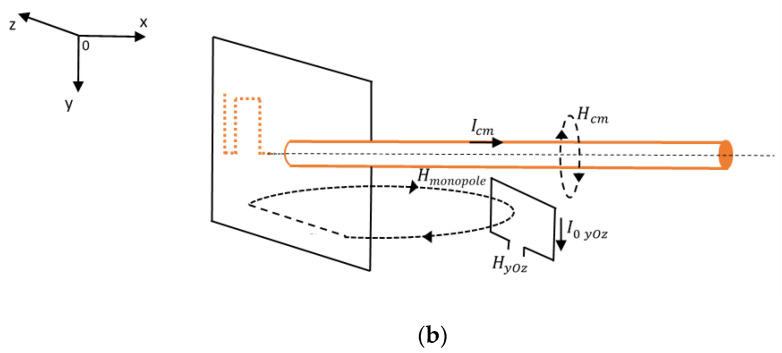
Magnetic field components through the PA: (**a**) *xOy* setup; (**b**) yOz setup.

For the meander line monopole antenna, the following measuring setups are defined:

(a) a *“xOy”* setup (Figure 20a)—the PA and the feed line are placed in the *xOy* plane; the PA will measure the total radiated field by the antenna–feeder set:
  (7) $$H_{xOy}=H_{cm}+H_{monopole\;}.$$
(b) a *“yOz”* setup (Figure 20b)—the PA and the ground plane are placed in the *yOz* plane; the PA will only measure the field radiated by the meander line monopole antenna:
  (8) $$H_{yOz}=H_{monopole\;}.$$

From (7) and (8), the field radiated by common mode currents can be extracted as:

(9) $$H_{cm}=H_{xOy}-H_{yOz\;}.$$

When applying the distance averaging method in order to reduce the effect of the common mode currents, the accuracy of the measurements is very important. Therefore, the method consists of measuring the transfer functions at *N* equally spaced distances between the two antennas, for both setups. The measurements are performed by mounting the antennas on a mobile platform that allows for the accurate positioning of the PA. 

The most important analytical relations [35] for applying our method express the average figures for the transfer functions (10), the contribution of the common mode current to the output current (11) and the magnetic field generated by the common mode currents (12):

(10) $${\bar{S}}_{21cm}=\overset{N}{\underset{k=1}{\sum }}\frac{d_{k}}{d_{0}}exp\;\left(jk_{0}d_{k}\right)\;S_{21\;cm}^{d_{k}},$$

(11) $${\bar{I}}_{0cm}=\frac{V_{g}\left|{\bar{S}}_{21cm}\right|}{2R_{0}},$$

(12) $${\bar{H}}_{cm}=\sqrt{\frac{R_{0}\;{\bar{I}}_{0\;cm}^{2}}{\eta A_{e}}\;}.$$

## 5. Results

### 5.1. Antenna Gain Measurements

**Figure 21:**
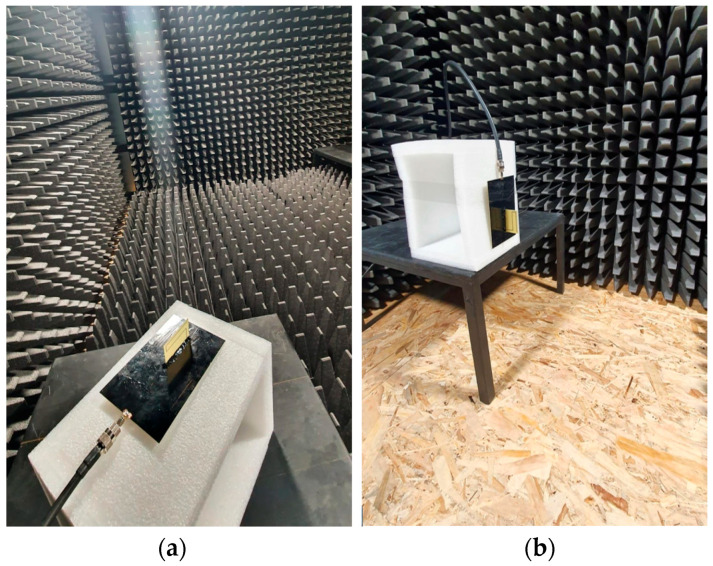
Measuring configuration for the input reflection coefficient: (**a**) with absorbers on the floor; (**b**) without absorbers on the floor.

The measurement configurations required for extracting the transfer function are presented in Figure 21. In order to compute the transfer function, as in (5), the reflection coefficient at the antenna input was measured with and without a reflector by simply keeping or removing the absorbers on the anechoic chamber floor.

**Figure 22:**
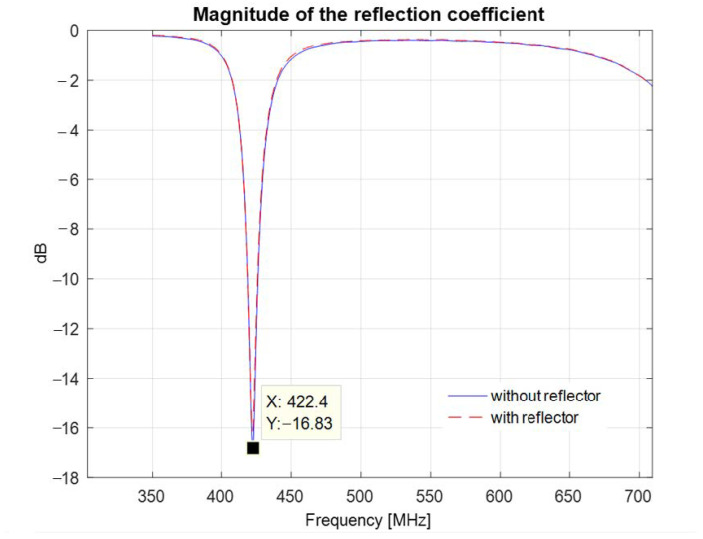
Magnitude of the reflection coefficient of the printed meander line monopole antenna with an input impedance matching circuit, measured with and without a reflector.

Figure 22 presents the magnitude of the reflection coefficient measured in the anechoic chamber with and without a reflector. The reflection coefficient exhibits a minimum of −16.83 dB at 422.4 MHz.

**Figure 23:**
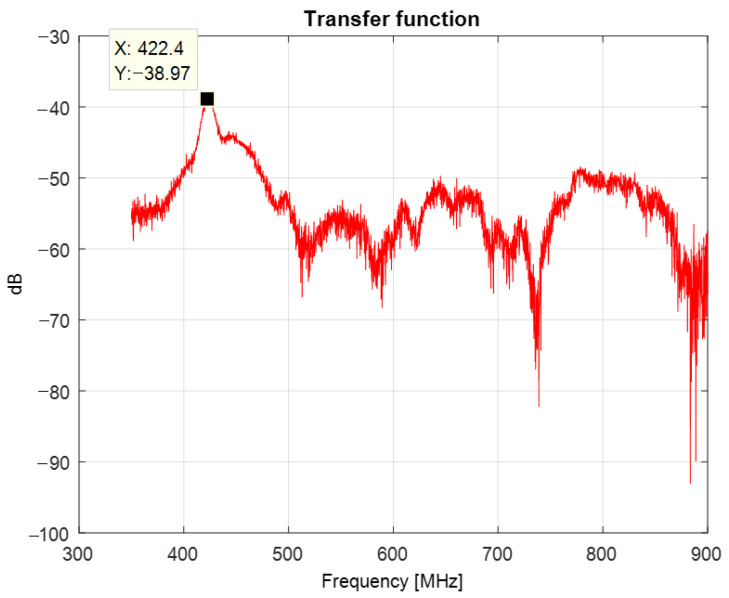
Transfer function of the system consisting of the antenna and its image.

Figure 23 presents the resulting transfer function of the system consisting of the antenna and its image. A maximum of −38.97 dB is observed at 422.4 MHz.

**Figure 24:**
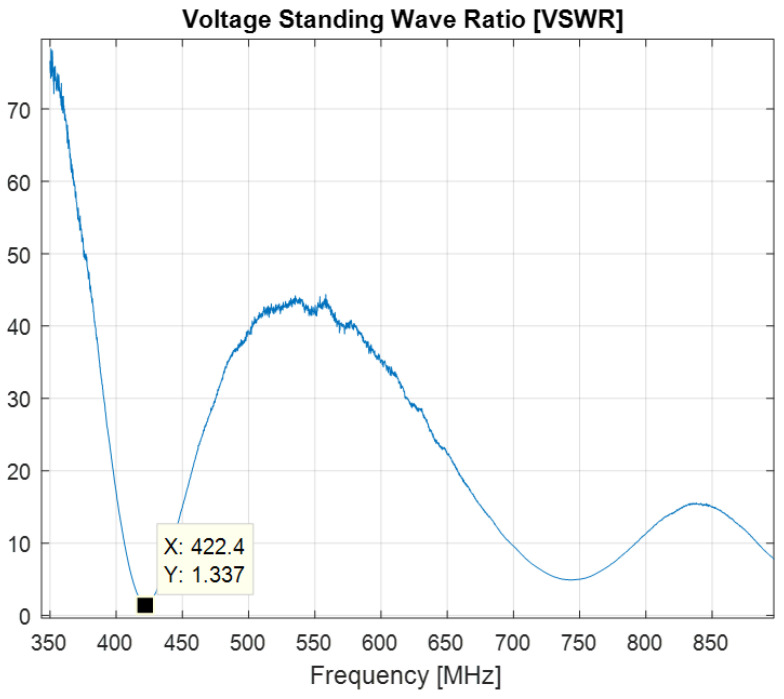
VSWR of the printed meander line monopole antenna with an input impedance matching circuit.

Figure 24 shows the VSWR as a function of frequency; a minimum of 1.33 is attained at 422.4 MHz.

**Figure 25:**
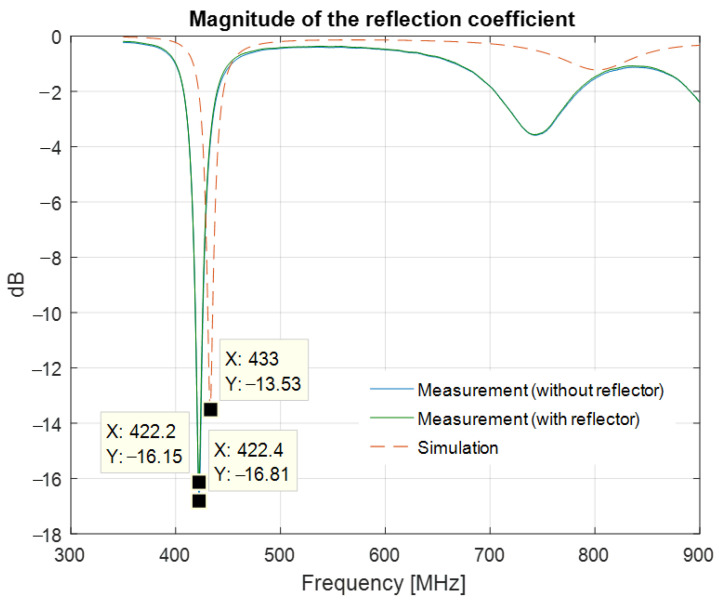
Magnitude of the reflection coefficient: measured and simulated results.

**Figure 26:**
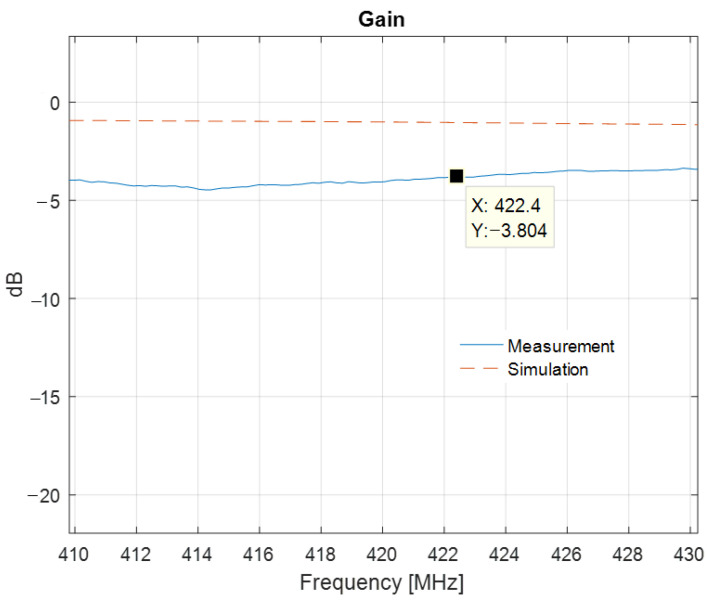
Gain of the printed meander line monopole antenna with an input impedance matching circuit: measured and simulated results.

Compared to the simulation results, the minimum of the measured reflection coefficient of the meander line monopole antenna is shifted down by 10.6 MHz. Figure 25 presents the $S_{11}$ parameter resulting from simulations and measurements. Figure 26 shows the variation in the gain over a bandwidth of 20 MHz around the transmitting frequency, as LoRa systems use narrowband transmissions.

The measured antenna gain at the transmission frequency, i.e., 422.4 MHz, is −3.80 dBi.

### 5.2. Radiation from Common Mode Currents

**Figure 27:**
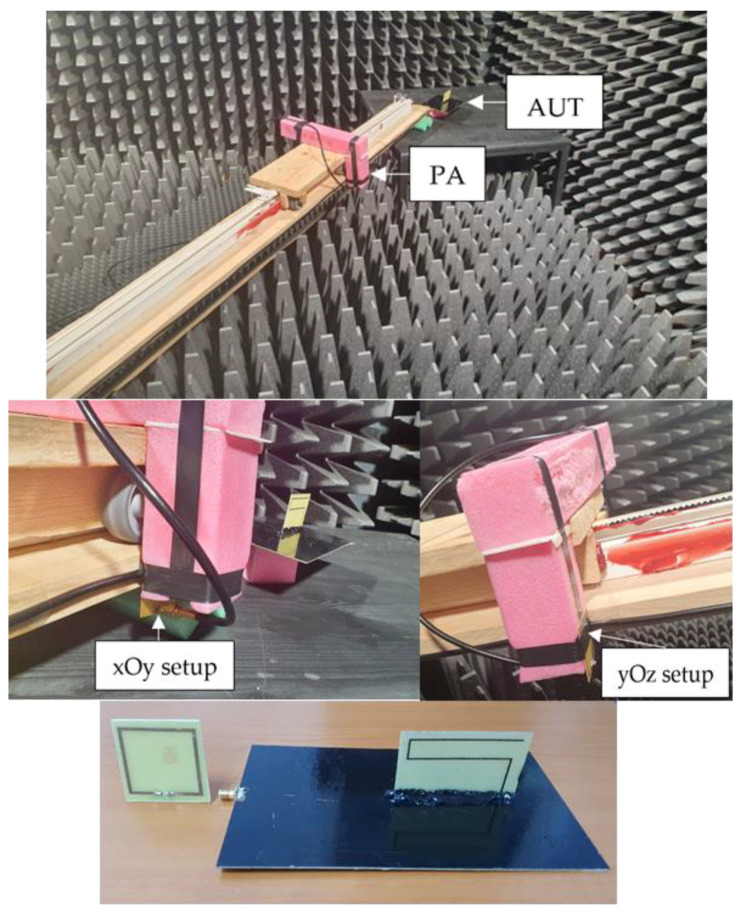
Measuring setup: mobile platform, *xOy* setup, *yOz* setup and antenna system details.

A two-antenna system for evaluating the effect of the common mode currents, consisting of the meander line monopole antenna as an AUT and a loop antenna as a PA, is presented in Figure 27. The PA and the AUT were connected to a vector network analyzer, and the measurements were performed in an anechoic chamber. 

**Figure 28:**
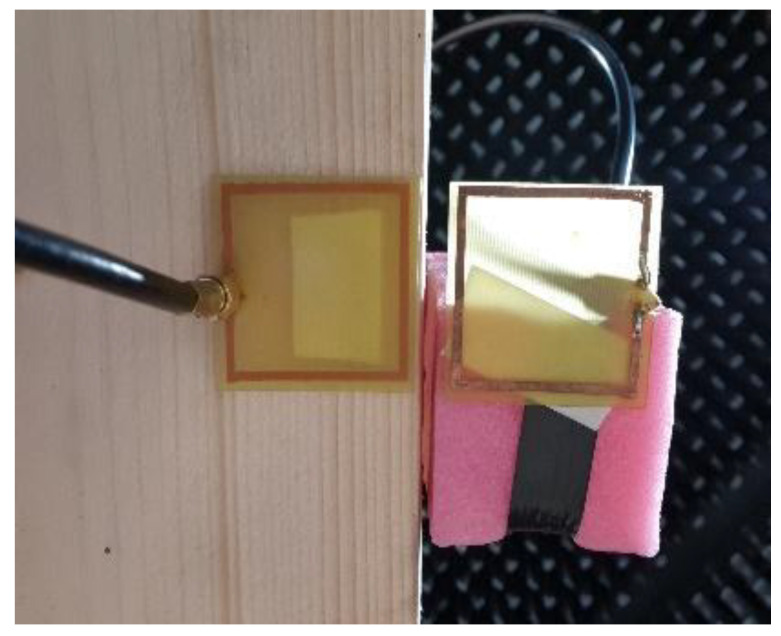
Loop antenna calibration.

Prior to evaluating the radiation originating from the common mode currents, the loop antenna used as a PA was first characterized in terms of effective area by using a two-identical antenna method [23] (Figure 28).

**Figure 29:**
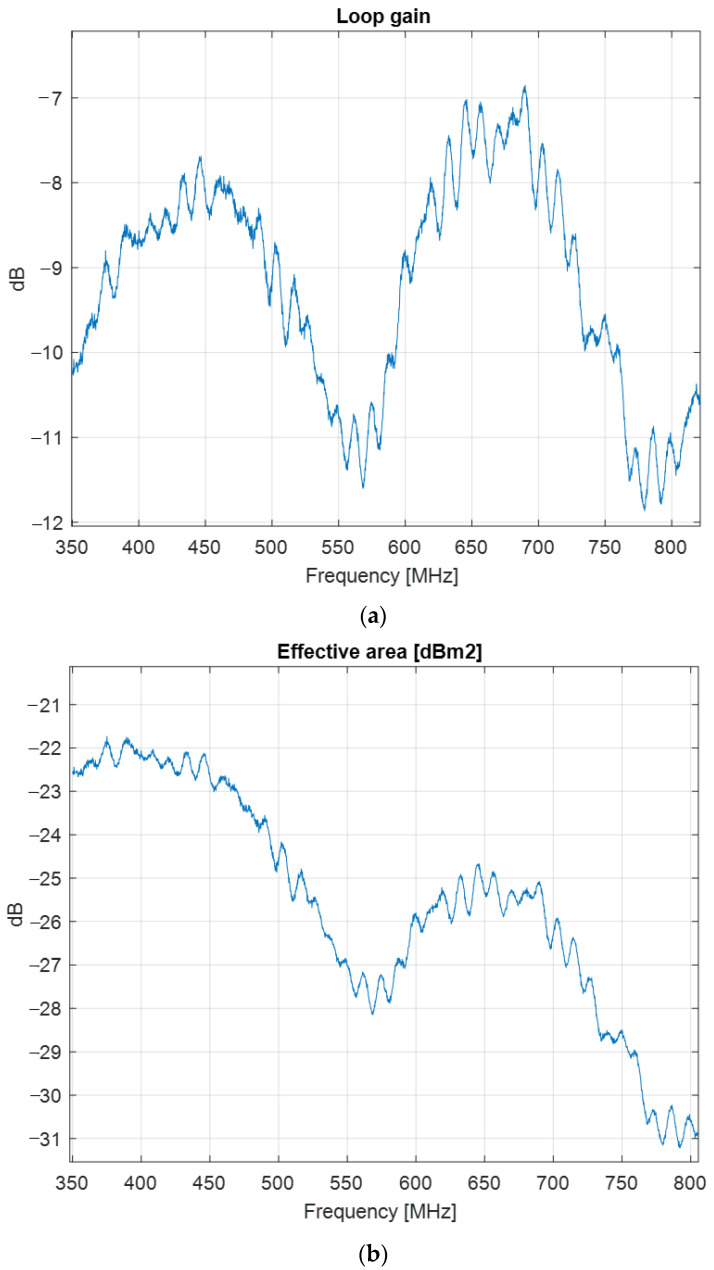
Loop antenna characteristics: (**a**) gain; (**b**) effective area.

The gain and the effective area of the loop antenna are presented in Figure 29.

The loop antenna was further used as a probe for both setups. For each setup, the measurements were performed at distances between 21 cm and 71 cm, with a pitch of 2 cm, and at frequencies between 300 MHz and 800 MHz. 

**Figure 30:**
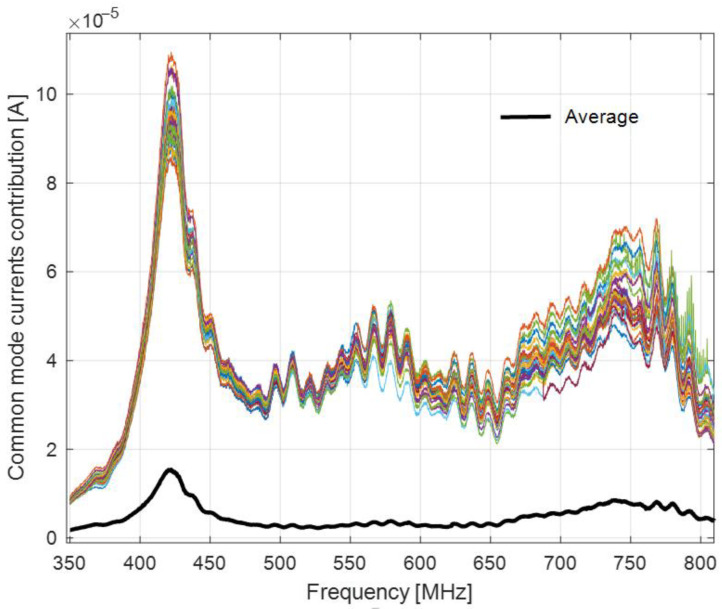
Contribution of the common mode current to the output current versus the distance-averaged figure.

Figure 30 presents the contribution of the common mode current and the average figure over the entire set of distances.

As Figure 30 shows, the contribution of the common mode current to the output current can be significantly reduced by applying the distance averaging method.

**Figure 31:**
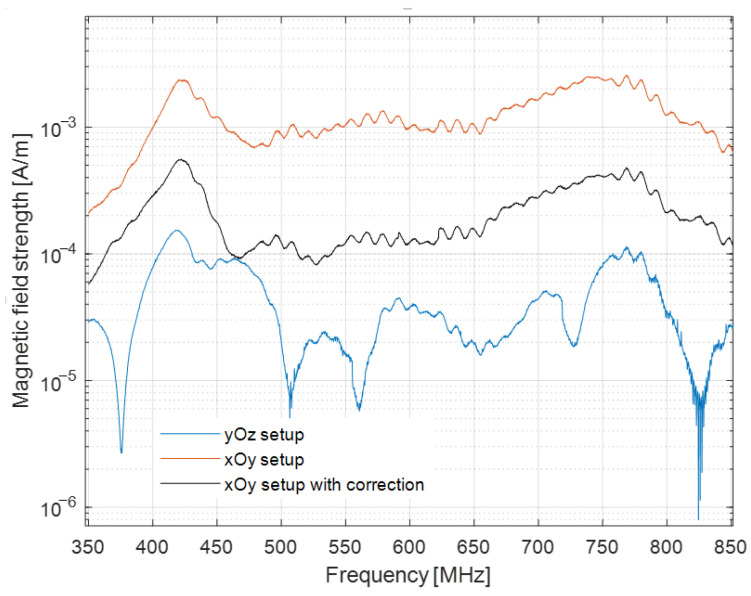
Magnetic field measured with the *xOy* setup, with and without correction, and the magnetic field measured with the *yOz* setup.

Figure 31 shows a comparison between the magnetic fields for both setups and the magnetic field for the *xOy* setup after applying the distance averaging method. 

It is revealed that applying the proposed method results in reducing the discrepancies between the magnetic field measured with the two setups.

**Figure 32:**
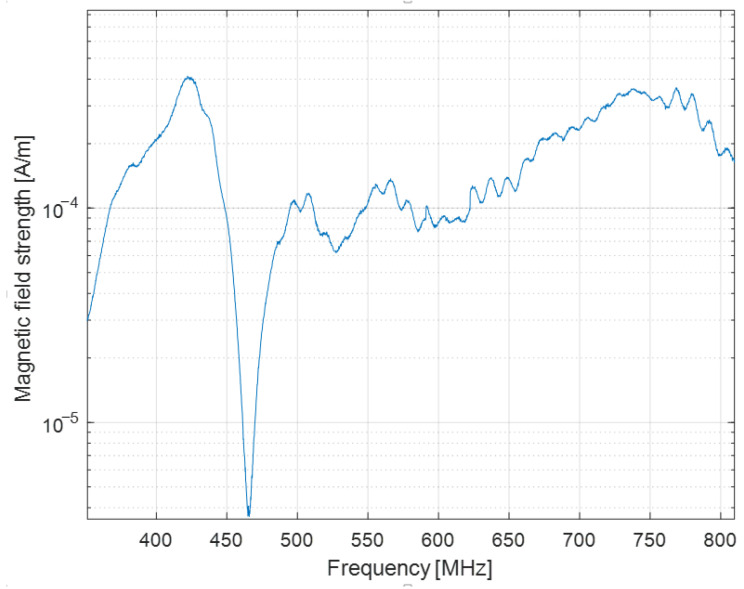
Magnetic field generated by common mode currents.

Figure 32 depicts the magnetic field strength after applying our differential approach, i.e., the magnetic field generated by the common mode currents.

At the resonant frequency, the antenna generates a magnetic field strength of $2\cdot 10^{-3}A/m$. Figure 32 displays the contribution of the common mode currents of $3\cdot 10^{-4}A/m$, which is much less than that of the field produced by the radiating element. 

### 5.3. Antenna Test in a Real Environment

**Figure 33:**
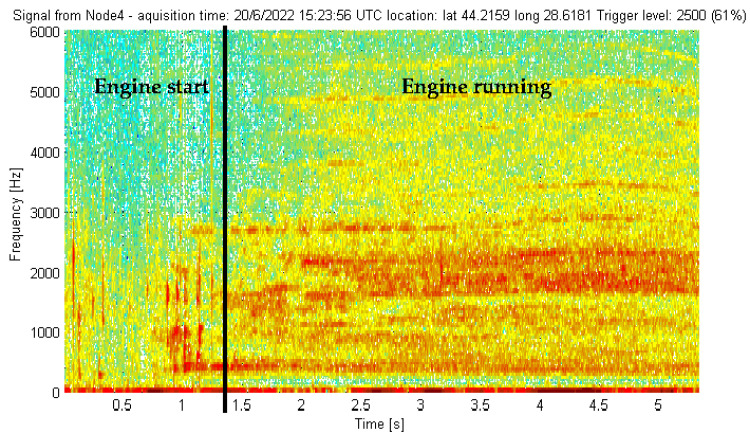
Signal picked up by a beacon in the presence of a motor boat.

**Figure 34:**
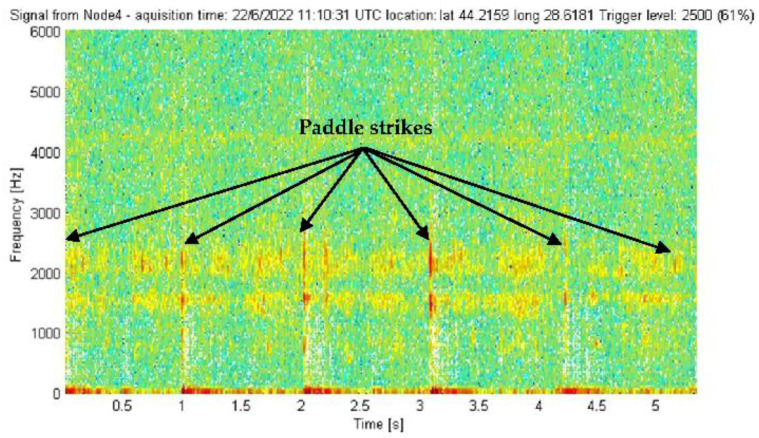
Signal picked up by a beacon in the presence of a paddle boat.

The experimental validation of the underwater acoustic monitoring system equipped with our meander line monopole antenna was carried out through the continuous monitoring of an aquatic area. The final version of the antenna was waterproofed by varnishing the radiator and the soldering point. The varnish layer does not impact the antenna performance, as the operating frequency is quite low. During the monitoring, some relevant events producing a triggering of the beacons could be recorded, e.g., motor boat incursions into the monitored area. Figure 33 shows the spectrogram for the entry of a motor boat into the detection area of a beacon. Figure 34 shows the spectrogram for a paddle boat incursion event. The detection range was in the order of tens of meters for the motor boat and of meters for the paddle boat, adjustable by the trigger threshold. Lowering the threshold increases the detection range. Obviously, the threshold value selection should also take into account the noise level of the specific deployment location in order to minimize the probability of false alarms. Extremely rough weather conditions may increase the noise level due to larger waves; however, the proposed system is intended only for inland waters such as lakes, canals, rivers and watercourses where waves are expected to be small to moderate. The optimum threshold value should be a compromise between the detection range (sensitivity), which demands a lower value, and the probability of false alarms, especially in bad weather conditions, which asks for a higher value. In our experiments, a value of 61% offered a good compromise. 

**Figure 35:**
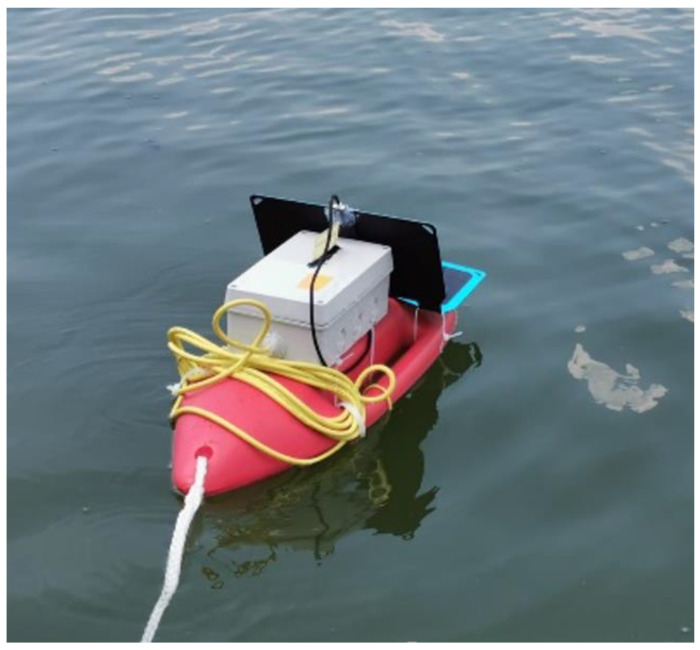
A beacon deployed in its operating environment.

Figure 35 shows a beacon deployed in its operating environment, a lake in the Danube Delta region.

Regarding the assessment of the transmission range, let us consider a node placed on a typical, 30 m-high communication tower. Since the beacon antenna is placed close the water surface, the maximum line-of-sight range will be

(13) $$r_{max}≅\sqrt{2hR},$$

where *h* is the tower height and *R* is the Earth’s radius. Under those circumstances, the maximum range would be 27.65 km. Our ongoing channel sounding measurements in a real operating environment show an excess pathloss of up to 14 dB compared to the free space. With the sensitivity figures given in the LoRa module specifications, the transmission range, by taking into account the excess pathloss, would be in the order of 100 km, which would be beyond the line-of-sight range. 

## 6. Conclusions

In this paper, we presented a compact meander line monopole antenna for equipping a network of floating beacons for underwater acoustic monitoring. The LoRa communication modules were operating at 433 MHz. The antenna design was optimized in terms of size and gain by using genetic algorithms and in terms of input matching by integrating a microstrip line circuit in the ground plane. In order to characterize the antenna, we proposed a single-antenna method for gain measurements and a distance averaging approach in order to assess the effect of the common mode currents. The proposed concept was successfully validated through simulation and measurements; a measured gain figure of −3.8 dBi together with a quasi-omnidirectional radiation pattern fully comply with the requirements for the communication system. Moreover, our design provides good input matching at the frequency of interest. A shift in the resonant frequency by 10.6 MHz, as well as a difference of 2.6 dB on the gain figure, can be noted between the simulated and measured results. The discrepancies are mainly due to the dispersion of the dielectric constant of the FR4 substrate and to the sharp variation of the gain around the resonant frequency. 

The full system was eventually tested in a real environment, therefore proving the reliability of the communication system including the new antenna design. 

Compared to commercial whip antennas for 433 MHz (generally known as ‘rubber duck’), which are basically quarter-wave monopoles with a physical height of about 17 cm, our antenna has a physical height of about 4 cm, and it is made of a small PCB. The manufacturing cost is lower than that paid for such a commercial monopole—typically less than one-quarter. It should be noted that both commercial monopoles and our antenna design need a ground plane. The gain of a monopole antenna highly depends on the ground plane size when both the ground length and width are less than 1.5λ, i.e., less than 1 m at 433 MHz. For our application, the ground plane is even electrically small, as it is 12 by 18 cm. The main lobe gain of the proposed antenna (which is around 1 dBi) is therefore not much lower than the gain of a quarter-wave whip antenna for the same ground plane size. 

Further work will focus on a dedicated radio wave propagation model, including the effect of the water surface, wind and waves.
